# Gene expression analysis indicates extensive genotype-specific crosstalk between the conjugative F-plasmid and the *E. coli *chromosome

**DOI:** 10.1186/1471-2180-6-80

**Published:** 2006-09-18

**Authors:** Bettina Harr, Christian Schlötterer

**Affiliations:** 1 Institut für Genetik, Universität Köln, Zuelpicher Strasse 47, 50674 Köln, Germany; 2 Institut für Tierzucht und Genetik, Veterinärmedizinische Universität Wien, Veterinärplatz 1, 1210 Wien, Austria

## Abstract

**Background:**

Plasmids are an important component of the bacterial genome, but the crosstalk between genes encoded on the chromosome and on the plasmid is still poorly understood.

**Results:**

We performed a large-scale survey for genes on the *E. coli *chromosome that are affected by the presence of the conjugative F-plasmid (crosstalk). The expression pattern of about 4% (107 genes) of the genes encoded by the chromosome was affected by the presence of the F-plasmid. Comparing two different *Escherichia coli *strains, MG1655 and DH5α, we found a strong host genotype-specific crosstalk of the host chromosome with the F-plasmid. About 88% of the genes affected by the presence of the F-plasmid showed a significant plasmid by host genotype interaction, i.e. the presence of the F-plasmid resulted in a different gene expression in the two host genotypes. Less than 12% of the genes showed an additive effect of gene expression, i.e. host genotype independent crosstalk between plasmid and host chromosome.

**Conclusion:**

We propose that epistatic effects also contribute to the maintenance of F-plasmids in natural populations.

## Background

Bacterial plasmids are extrachromosomal, non-essential DNA elements, which can replicate autonomously [1,2]. Most plasmids are smaller than bacterial chromosomes and some plasmids have the capacity to move between different host species, leading to intra- and inter-specific gene transfer [3,4].

The co-existence of the plasmid and the bacterial chromosome in the same cell raises interesting questions. Probably the best studied is how plasmids persist in natural populations. It has been proposed that plasmids are parasites [5] or mutualists [6], conferring a selective advantage to their host. Eberhard [2] suggested that plasmids carry genes, which are only required under certain rare circumstances. When needed they can be transferred to other cells, but when not required only a small number of cells carry the plasmid. According to this view plasmids are a kind of 'lending library' that deliver genes when required.

While the above mentioned hypotheses do not require crosstalk between plasmid and the host chromosome, some studies using experimental evolution showed that the host chromosome and the plasmid co-evolve to reduce the cost of F-plasmid carriage [7,8]. Nevertheless, the extent of crosstalk between host chromosome and plasmids is not known.

In this study, we compare the gene expression pattern of the *E. coli *chromosome in the presence and absence of the conjugative F-plasmid to understand how the presence of the plasmid affects gene expression of the chromosome. Using two different *E. coli *strains, we find dramatic differences in the expression of host genes, indicating a highly genotype specific crosstalk between plasmid and chromosome.

## Results

4374 genes are represented on the Affymetrix chip (not counting the intergenic regions), 2698 of these were expressed in at least one of the four different genotypes (two hosts, each in the presence or absence of the plasmid). Our experimental set-up was designed to disentangle the contribution of genetic background of the host from changes in gene expression induced by F-plasmid.

**Figure 1:**
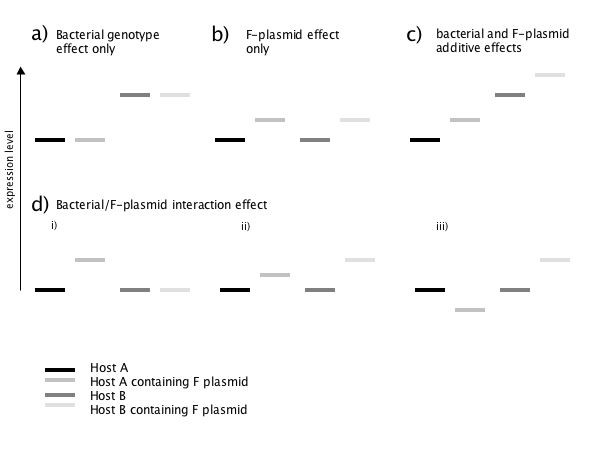
**Possible effects of bacterial strain and F-plasmid in a 2 × 2 analysis of variance (ANOVA)**. a) -c): cases with no interaction effect. d): examples of some possible interaction effects.

We distinguish four groups of genes: "bacterial genotype effect only" (Fig. 1a), "F-plasmid effect only" (Fig. 1b), "bacterial and F-plasmid additive effects" (Fig. 1c), and "bacterial/F-plasmid interaction effect" genes (Fig. 1d).

### a) Bacterial genotype effect only

We identified a total of 216 genes with a host-specific gene expression independent of the presence of the F-plasmid ("bacterial genotype effect only" genes, [see Additional file 1]). After accounting for genes located in the same operon we identified 141 unique "regulatory units" (i.e. sets of genes that are co-regulated due to their location in the same operon). 57 regulatory units were up-regulated in DH5α and 84 regulatory units were up-regulated in MG1655. Interestingly, the host-specific genes in the two strains fell into functionally different classes. All 11 amino acid synthesis regulatory units with host-specific gene expression were up-regulated (~6-fold) in MG1655 (Table 1). Genes affecting nucleotide biosynthesis, carbon utilization and fatty acid synthesis were also up-regulated (between ~4 and 10 fold) in MG1655. The opposite pattern was found for flagellum and chemotaxis genetic regulatory units, which were strongly up-regulated in DH5α (between ~24 and ~28-fold). Similarly, regulatory units with prophage- or transposon-related functions were more highly expressed in DH5α.

### b) F-plasmid effect only

An "F-plasmid effect only" gene is a gene that is expressed in response to the presence of the F-plasmid but without having any strain-specific effects. As expected, all 10 F-plasmid encoded genes that were represented on our microarray were strongly expressed in F-plasmid containing cells, but not expressed (i.e.: had signal intensities below the detection limit and were called "Absent" by the Affymetrix software) in F-plasmid free cells. Nine out of these 10 F-plasmid encoded genes were "F-plasmid effect only" genes (Table 2) while one F-plasmid encoded gene showed an interaction effect with the host genotype (see below). All "F-plasmid effect only" genes show an increase in expression level in the presence of the F-plasmid. The difference in expression level of F-plasmid encoded proteins between F-plasmid free cells (where expression level equals background signal intensity on the chip) and F-plasmid containing cells was on average 40-fold with a maximum change in expression level of the *traD *gene of 140-fold.

There are seven genes encoded on the bacterial chromosome that were at least two-fold differentially expressed in cells with and without F-plasmid. On average these are up-regulated by ~3.3-fold.

### c) Bacterial and F-plasmid additive effects

**Figure 2:**
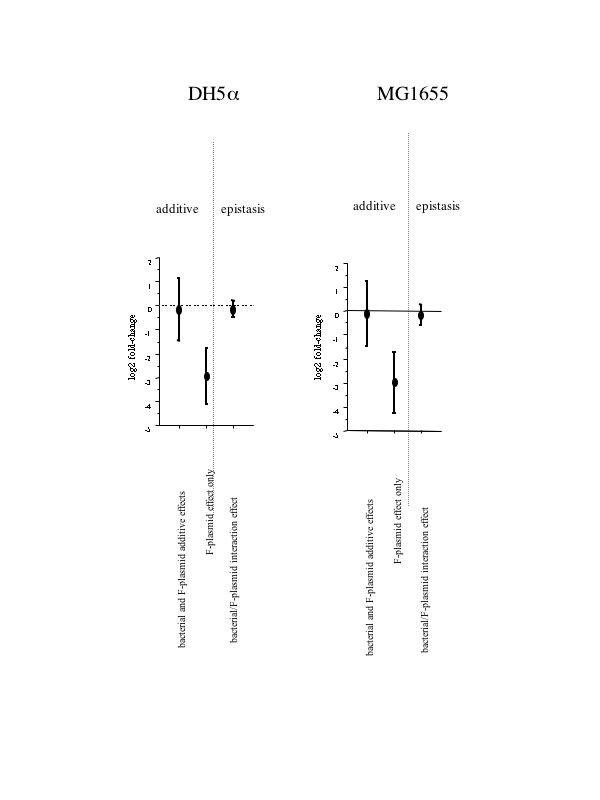
**Direction of expression differences**. The mean (± 2 s.e.) of log_2 _transformed fold-change in expression level in response to the absence of the F-plasmid are plotted for two different *E. coli *strains, DH5α and MG1655. While "plasmid effect only" genes showed the expected change in one direction only (expression in the presence of the F-plasmid), the "bacterial and plasmid additive effects only" genes and "bacterial/plasmid interaction effect" genes were unbiased (i.e.: the mean change across genes was close to zero).

We identified six genes which had different expression levels in the two hosts and which also had an effect induced by the presence of the F-plasmid, but without any interaction effect (Table 3). In contrast to "F-plasmid effect only" genes, an equal number of genes are up-regulated and down-regulated in the presence of the F-plasmid  (Table 3). Furthermore, there was no net change in expression intensity (Figure 2).

### d) Bacterial/F-plasmid interaction effect

Genes falling into this category show either a significant effect of the F-plasmid in only one host, or have disproportional effects across the two hosts. In total, 95 interaction genes (82 regulatory units) were identified. 25 genes (20 regulatory units) had a significant difference in expression levels between MG1655 and MG1655F but not between DH5α and DH5αF. 49 genes (47 regulatory units) with a host-specific F-effect were detected in DH5α but not in MG1655. 20 genes (15 regulatory units) showed an F-effect in opposite direction in the two hosts [see Additional file 2]. One gene (the F-plasmid encoded *Protein-D *(*resD*)) was up-regulated in the presence of the F-plasmid in both genetic backgrounds but the magnitude of change was twice as strong in MG1655 than in DH5α. Similar to genes with "bacterial genotype effect only" or "F-plasmid effect only", interaction genes were also equally likely to be up- or down-regulated in the presence of the F-plasmid (Table 4) and no net change in expression intensity was noted (Fig. 2).

In contrast to the "bacterial genotype effect only" genes, a functional classification of the "bacterial/F-plasmid interaction effect" genes provided no clear pattern (Table 4). Among the genes with a host-specific response to the F-plasmid we found genes with functions in chaperoning and carbon utilization.

## Discussion

In our experiments 323 genes (out of 2698 expressed genes) located on the *E. coli *chromosome show significant differences in gene expression. 216 (67%) of these 323 genes differed in expression between the two *E. coli *genotypes DH5α and MG1655 and this difference was not affected by the presence of the F-plasmid. Based on the significance level of 0.05 used in our study, only 135 genes would be expected to show 'significant' differences by chance alone. In our study, however, we observed significantly more differently expressed genes than expected by chance (P < 0.000001, binomial test), confirming a significant biological effect. Note that the binomial test is conservative, as we involved an additional filtering step to include only genes with at least 2-fold expression difference (see material and methods). Further support for the reliability of our expression analysis is provided by the recovery of the well-known difference between both strains in the activity of the *lac *operon, which is inactive in DH5α (see appendix). This high number of strain specific differences in gene expression pattern is consistent with previous reports that also found large differences among *E. coli *strains [9,10].

About half of the strain-specifically expressed genes were not detectably expressed in the other strain (i.e. genes not expressed in DH5α were expressed in MG1655 and vice versa). We did not perform genomic hybridization to test the hypothesis that some of the changes in expression level are due to the absence of the respective gene, as the arrays had been constructed based on the sequence of the strain MG1655. As we also found genes expressed in DH5α but not in MG1655 we consider that gene deletions are not a major factor shaping the difference in gene expression between the two strains. This is supported by some recent work showing that gene content is very similar among related bacterial strains (for example strain W3110, a close relative of MG1655 lacks only 80 (1.9%) of MG1655's ORF's [11]). Nevertheless, even when only genes expressed in both strains were considered (i.e. 2243 genes), a statistically significant excess of differentially expressed genes (130) could be detected (P = 0.047, binomial test).

Of the 107 chromosomal genes that were affected by the presence of the F-plasmid we found less than 12% (13 genes) with purely additive effects across strains. The majority of genes showed a significant interaction between strain and plasmid in their expression. Hence, despite the fact that we used an F-plasmid with an identical genotype in our experiments, most of the genes showed a response to the presence of the F-plasmid that depended on the genotype of the host chromosome. The abundance of significant interaction effects between strain and plasmid gene expression implies that epistasis (i.e. genotype-specific crosstalk) is generally common. By contrast, of the 10 F-plasmid encoded genes that were represented on the chip, nine showed additive effects and were not significantly influenced by the host genotype. Even though the number of F-plasmid encoded genes is small, this difference is statistically highly significant (p < 0.001, Fisher's exact test), implying large differences between F-plasmid encoded and chromosomal genes. This result is not affected by the significance level used for the identification of significant genes (i.e. ANOVA P-value of 0.005 provided similar results, data not shown). The almost complete absence of interaction effects for F-plasmid encoded genes is not unexpected, as these genes are essential for the F-plasmid associated phenotype (e.g. formation of sex-pili). Thus, the F-plasmid, which could easily move among cells [4], requires a set of genes that remains functional in the genetic background of different host cells.

## Conclusion

Our results of a strong host genotype dependent crosstalk could potentially have important evolutionary implications. As the same plasmid results in a contrasting gene expression in different host genotypes, the cost of carriage is likely to depend on both the host genotype and the environment. Hence, it is conceivable that this host genotype specific crosstalk also results in fitness differences, i.e. the cost of carriage may vary among host genotypes. More host genotypes, in particular more diverged ones, need to be analyzed in a range of environments to determine if the epistatic interactions observed in our study could contribute to the maintenance of F-plasmids in natural populations.

## Methods

### Strains used

The two laboratory strains of *E. coli *used were DH5α ([F-Φ80d*lac*ZΔM15Δ(lacZYA-argF) U169 *end A1 recA 1 hsdR17*(r_k_-m_k_+) *deo*R *thi-*1 supE44 λ-*gyr*A96 *rel*A1]) and MG1655 (obtained from F. Blattner). The sequenced F-plasmid (GenBank Accession number: AP001918, obtained from Gen-ichi Sampei) was added to both strains by conjugation. Altogether four different genotypes were investigated: DH5α containing an F-plasmid, DH5α without an F-plasmid, MG1655 containing an F-plasmid and MG1655 without an F-plasmid. The identity of strains was confirmed on IPTG/X-Gal LB plates (MG1655 yields blue colonies while DH5α colonies are white) and by F-plasmid specific PCR using primers complementary to the F-plasmid encoded traU gene (5' gtc ttc ctg ggt gaa tga tg 3' and 5' gat gat gtc gta tcc ctg act g 3').

### Culture conditions

Replicate cultures for each genotype were inoculated from single colonies in 5 ml LB medium (0.2 mg/ml Ampicillin) and grown over night at 37°C. 500 μl of each overnight culture was used to inoculate 50 ml fresh LB medium. These cultures were grown at 37°C and cells were harvested in early log phase corresponding to an OD_600 _of 0.4. The identical batch of broth was used throughout the entire experiment.

### RNA extraction and hybridizations

1 ml early log phase culture was stabilized with 2 vol. RNAprotect Bacteria Reagent (Qiagen Cat# 76506) according to the manufacturers protocol. RNA was extracted with the MasterPure™ RNA Purification Kit obtained from Epicenter (Cat # MCR85102) following the manufacturer's protocol. To reduce background on the chip half of the recovered total RNA (12.5 μg) was subjected to a ribosomal RNA removal procedure using the MICROB *Express*™ Bacterial mRNA Purification Kit (Ambion. Cat# 1905). rRNA-free and rRNA-containing samples were then pooled and total, fragmented RNA was 3' end-labeled according to the Affymetrix protocol. Each sample was hybridized to an Affymetrix *E. coli *Antisense Array (Cat# 900381) following the manufacturer's protocol. Patterns of hybridization were detected with an Affymetrix scanner. Each genotype was replicated once so that two replicates per genotype could be analyzed.

### Data analysis

#### Selection of genes with differences in expression level

Raw signal intensities were analyzed according to the standard implementation of the Affymetrix Microarray Suite (MAS) 5.0 software, which summarizes expression values based on perfect match and mismatch probes. Signal intensities generated by the Affymetrix software were log_2 _transformed. All genes not expressed in at least one of the genotypes were removed. Specifically, within one genotype signal intensities of both replicates had to be > 250 or alternatively had to be called "Present" by the Affymetrix software. To identify genes significantly affected by the presence of the F-plasmid in the two different host genotypes we applied an analysis of variance (or "ANOVA" model). The ANOVA model examines the association between nominal predictor variables and a continuous outcome variable (e.g., gene expression). Since in our case we have two nominal predictor variables (e.g., "strain" and "with and without F-plasmid"), we used a two-way ANOVA [12]. The two-way ANOVA was performed on the log_2 _transformed signal intensity of each gene with bacterial genotype (DH5α or MG1655) and presence of F-plasmid as predictor variables (also called "factors").

We scored a gene as having a main effect or interaction effect if that effect showed statistical support (P < 0.05) and if the magnitude of the effect was at least two-fold. In addition, the interaction effect was scored as present if P < 0.05 and the effects of the plasmid went in opposite directions in the different bacterial genotypes.

Genes were divided into four categories "bacterial genotype effect only", "F-plasmid effect only", "bacterial and F-plasmid additive effects" and "bacterial/F-plasmid interaction effects". In the "bacterial genotype effect only" category, only the main effect of the bacterial genotype was scored as being present (Figure 1a). If only the main effect of the plasmid was present, we labeled this gene as "F-plasmid effect only" (Figure 1b). If both main effects were present, then the genes were further divided by whether an interaction effect was present: "bacterial and F-plasmid additive effects" was the group without interaction effect (Figure 1c) and "bacteria/F-plasmid interaction effects" was chosen in the presence of an interaction effect (Figure 1d, i.–iii.).

The presence of the F-plasmid could have either an additive effect (Figure 1b, c) or a non-additive effect with a genotype by plasmid interaction (Figure 1d, i.–iii.). Significant bacterial genotype by F-plasmid interactions were identified by pairwise HSD (Tukey's Honest Significant Difference) tests. The HSD test conducts multiple comparisons at all possible pairwise comparisons of the 4 genotypes, simultaneously correcting for multiple testing. Comparisons for which the 95% confidence intervals do not overlap zero are here considered significant. Depending on the type of interaction, we either conditioned on genes, which showed a significant difference in signal intensities between DH5α and DH5αF but not between MG1655 and MG1655F (Figure 1d i.) or genes, which are significantly affected by the F-plasmid in both backgrounds but to a different extent (Figure 1d, ii.). The third group of interaction effects consisted of genes, which were affected in the opposite direction (Figure 1d, iii.).

All statistical analyses were performed using Perl scripts (available from the authors upon request) and the statistical language *R*. Affymetrix MAS5.0 normalized signal intensities were submitted to GEO [13] and are available under the following Series ID: GSE1154.

### Functional grouping of candidate genes

Candidate genes were classified into functional groups according to the EcoCyc database (Encyclopedia of *Escherichia coli *Genes and Metabolism [14]) specifications. Candidate genes located in the same operon (as inferred from RegulonDB [15] were summarized as single "regulatory unit". If genes were members of operons and the genes within one operon had different functions each of them was weighted equally. For example, an operon with one gene functioning in carbon utilization and another one functioning as a chaperone was treated as 0.5 carbon utilization and 0.5 chaperoning. Single genes with multiple functions were disregarded, as their classification would have been ambiguous. Functional groups were only assigned if at least two different candidate genes or regulatory units could be assigned to that group.

## Competing interests

The author(s) declare that they have no competing interests.

## Authors' contributions

BH and CS designed the experiment and wrote the manuscript. B. Harr performed the experiments and data analysis.

## Supplementary Material

Additional File 1 Bacterial genotype only genes. List of genes that are differentially expressed between DH5α and MG1655. Click here for file

Additional File 2 Bacterial/F plasmid interaction genes. List of genes that show an interaction effect between host genotype and F plasmid. Click here for file

## Figures and Tables

**Table 1:** Functional groups of genes differentially expressed between hosts.

Gene functional type	MG1655	Fold-change^#^	DH5α	Fold-change^#^
Amino acid biosynthesis*	11	5.93^a)^		
Carbon utilization	8.5	10.23^a)^	1	24.10^b)^
Chemotaxis			4.5	24.33^b)^
Energy production	2.5	3.00^a)^	4	3.02^b)^
Transposon-related	4.5	3.90^a)^	9	172.50^b)^
Fatty acid biosynthesis	4	3.95^a)^		
Flagellum			9.5	28.94^b)^
Information transfer	8	3.24^a)^	5	9.37^b)^
Metabolism general	3	107.20^a)^	5	3.13^b)^
Unknown	18.5	3.30^a)^	8	8.77^b)^
Nucleotide biosynthesis	10.5	6.40^a)^	2	7.92^b)^
Pilus (type I)	2	3.22^a)^		
Transport	2.5	2.49^a)^	2	2.48^b)^

Number and average fold-change of genes, up-regulated in DH5α relative to MG1655 (right two columns) and number and average absolute fold-change of genes up-regulated in MG1655 relative to DH5α (left two columns).

*affected amino acids: isoleu/val, gly, try, phe, arg, asp, tyr, thr/ser.

# average among regulatory units within given functional group,

a): higher expression in MG1655 b): higher expression in DH5α.

**Table 2:** "F-plasmid effect only" genes: similar effects of F-plasmid in both bacterial backgrounds

	Fold-change			
gene	F/nonF (MG1655)	F/nonF (DH5α)	P (F-effect)	function
*b0373*	2.72	3.49	0.00048	IS3 element
*b1171*	4.01	4.03	0.00543	ORF
b2254*	5.20	2.12	0.00323	putative sugar transferase
b2255*	4.56	2.53	0.0068	putative transformylase
*osmB*	2.06	3.92	0.0041	osmotically inducible lipoprotein
***Protein-A***	77.75	80.04	0.00001	*sopA*
***Protein-B***	16.18	12.71	0.00405	*sopB*
***Protein-C***	6.76	9.96	0.00004	*repC *(replication protein)
***Protein-E***	11.49	19.49	0.00553	*repE *(replication initiation protein)
***Protein-G***	36.00	15.14	0.00005	*ccdB *(*letD*) post-segregational killing
***Protein-H***	26.80	26.87	0.00001	*ccdA *(*letA*) suppression of *ccdB*
***traD***	168.46	127.67	0.00008	DNA transport
***traI***	11.57	15.46	0.00003	oriT nicking and unwinding
***trbH***	24.69	24.78	0.00015	unknown
*metU*	-4.09	-2.56	0.02676	Methionine tRNA; duplicate gene
*yqgB*	-2.60	-2.49	0.02466	ORF

* genes are members of the same operon.

Negative fold-change values indicate down-regulation in F-plasmid free cells.

Genes given in bold are coded on the F-plasmid. Note that the genes were called absent, but rather than setting the expression to zero, we used the hybridization signal detected to calculate the ratio between cells with and without F-plasmid.

**Table 3:** "Bacterial and F-plasmid additive effects": genes whose expression level differs depending on bacterial background and whether or not they harbor an F-plasmid

	Fold-change						
Gene	F vs. nonF (MG1655)*	F vs nonF (DH5α)*	MG1655 vs. DH5α^#^	P (F-effect)	P (strain-effect)	Function	Process
*b1722*	3.03	2.36	-2.03	0.005	0.012	Conserved-Hypothetical-ORF	
*flhD*	2.34	2.61	-5.22	0.002	0.0002	regulator of flagellar biosynthesis, transcriptional initiation factor	Motility, chemotaxis, energytaxis (i.e. aerotaxis, redoxtaxis)
*leuQ*	2.78	2.19	3.50	0.050	0.022	Leucine tRNA	
*osmY*	-2.98	-2.11	3.24	0.003	0.001	hyperosmotically inducible, periplasmic protein	
*tra5_3*	-2.53	-2.77	2.36	0.005	0.007	IS3 putative transposase	
*ykfE*	-3.62	-4.84	2.13	0.003	0.023	ORF	

*: Negative values indicate down-regulation in F-plasmid free cells.

#: Negative values indicate down-regulation in MG1655.

**Table 4:** Regulation of "bacterial/F-plasmid interaction effect" genes

*MG1655*		*DH5α*		
down	up	down	up	Functional class
1	3	2.5	2	Amino-acid biosynthesis
9	0.67	0.33	5	Carbon utilization
0	3.83	2.17	0	Chaperone
1	1.33	0.33	4	Energy metabolism
1	0	3	6	Information transfer
2	3.33	0	2	Metabolism general
0	0	1	2	Murein biosynthesis
1	5	11	9	Unknown
1	0	2	1	Nucleotide metabolism
0	0	1.5	2	Ribosome
0	1	1.5	0	Transport

down: down-regulated in F-plasmid containing strain.

up: up-regulated in F-plasmid containing strain.
