# Supplementary material for: Gene expression analysis indicates extensive genotype-specific crosstalk between the conjugative F-plasmid and the E. coli chromosome
Source: BMC Microbiol. 2006 Sep 18;6:80. doi: 10.1186/1471-2180-6-80 (PMC1590023; doi:10.1186/1471-2180-6-80)
Supplement: Additional File 1 — Bacterial genotype only genes. List of genes that are differentially expressed between DH5α and MG1655. [file 1471-2180-6-80-S1.doc]

Additional file 1

“Bacterial genotype effect only” genes: differentially expressed between DH5α and MG1655. Genes are ordered according to whether they have higher expression level in MG1655 (upper part of table) or higher expression level in DH5α (lower part of table).

| Gene | *MG1655* Gene Identifier | fold change | operon no. | ANOVA P-value (strain) | functional class |
| --- | --- | --- | --- | --- | --- |
| *More highly expressed in MG1655* | | |  |  |  |
| pheA | *b2599* | 9.91 | OP24 | 8.51E-04 | amino acid |
| pheL | *b2598* | 19.75 | OP24 | 1.08E-02 | amino acid |
| ivbL | *b3672* | 2.03 | OP27 | 2.59E-02 | amino acid |
| ilvB | *b3671* | 2.53 | OP27 | 2.33E-03 | amino acid |
| tnaB | *b3709* | 2.43 |  | 4.55E-03 | amino acid |
| argT | *b2310* | 2.64 |  | 1.59E-03 | amino acid |
| pheM | *b1715* | 2.69 |  | 8.86E-03 | amino acid |
| ygjU | *b3089* | 2.76 |  | 3.73E-04 | amino acid |
| tyrP | *b1907* | 3.06 |  | 7.29E-03 | amino acid |
| glyA | *b2551* | 3.56 |  | 3.41E-04 | amino acid |
| gadB | *b1493* | 3.87 |  | 1.98E-02 | amino acid |
| cstC | *b1748* | 5.11 |  | 7.21E-04 | amino acid |
| asnC | *b3743* | 22.05 |  | 5.99E-04 | amino acid |
| araE | *b2841* | 2.04 |  | 1.17E-02 | carbon |
| kdgK | *b3526* | 12.80 |  | 9.15E-06 | carbon |
| kduI | *b2843* | 52.44 |  | 3.25E-05 | carbon |
| rbsB | *b3751* | 3.86 | OP30 | 2.21E-04 | carbon |
| rbsK | *b3752* | 5.42 | OP30 | 2.39E-04 | carbon |
| rbsD | *b3748* | 5.60 | OP30 | 2.28E-03 | carbon |
| rbsA | *b3749* | 5.96 | OP30 | 5.72E-05 | carbon |
| rbsC | *b3750* | 6.18 | OP30 | 5.79E-04 | carbon |
| yjcW | *b4087* | 2.58 | OP34 | 3.84E-02 | carbon |
| yjcX | *b4088* | 3.16 | OP34 | 7.48E-04 | carbon |
| rpiR | *b4089* | 4.27 | OP34 | 1.18E-03 | carbon |
| lldP | *b3603* | 4.31 | OP52 | 9.41E-04 | carbon |
| yiaE | *b3553* | 2.04 |  | 1.02E-02 | carbon |
| glcC | *b2980* | 2.40 |  | 9.26E-04 | carbon |
| melB | *b4120* | 5.90 |  | 7.07E-04 | carbon |
| fhuF | *b4367* | 2.03 |  | 1.41E-03 | chaperone |
| edd | *b1851* | 3.61 | OP12 | 1.02E-04 | energy |
| eda | *b1850* | 3.88 | OP12 | 1.55E-03 | energy |
| lldD | *b3605* | 7.12 | OP52 | 1.25E-02 | energy |
| glpR | *b3423* | 2.26 |  | 4.44E-03 | energy |
| entE | *b0594* | 2.15 | OP8 | 2.28E-02 | enterobactin |
| entC | *b0593* | 2.44 | OP8 | 1.20E-02 | enterobactin |
| cvpA | *b2313* | 6.37 | OP51 | 2.53E-05 | extrachromosomal |
| fepA | *b0584* | 2.03 |  | 1.96E-02 | extrachromosomal |
| cirA | *b2155* | 2.44 |  | 4.03E-03 | extrachromosomal |
| tsx | *b0411* | 2.80 |  | 4.62E-02 | extrachromosomal |
| insA5 | *b1894* | 5.11 |  | 8.74E-03 | extrachromosomal |
| b2341 | *b2341* | 2.48 |  | 8.34E-03 | fatty acid |
| mhpR | *b0346* | 2.90 |  | 3.19E-02 | fatty acid |
| fadB | *b3846* | 4.55 |  | 4.01E-05 | fatty acid |
| acs | *b4069* | 5.86 |  | 3.49E-04 | fatty acid |
| yrdD | *b3283* | 2.32 |  | 2.07E-02 | gyrase |
| ygeA | *b2840* | 2.40 |  | 1.13E-02 | information |
| hdeA | *b3510* | 6.22 | OP26 | 6.60E-04 | information |
| ibpA | *b3687* | 2.80 | OP28 | 1.60E-02 | information |
| ibpB | *b3686* | 4.76 | OP28 | 1.65E-03 | information |
| yhiX | *b3516* | 2.16 |  | 4.19E-02 | information |
| dps | *b0812* | 2.16 |  | 4.44E-02 | information |
| evgA | *b2369* | 2.20 |  | 1.44E-02 | information |
| bfr | *b3336* | 2.88 |  | 4.05E-04 | information |
| soxS | *b4062* | 3.83 |  | 4.32E-03 | information |
| lacZ | *b0344* | 22.86 | OP5 | 2.60E-05 | lac-operon |
| lacA | *b0342* | 26.42 | OP5 | 8.24E-05 | lac-operon |
| lacY | *b0343* | 32.82 | OP5 | 6.77E-05 | lac-operon |
| kduD | *b2842* | 314.03 |  | 1.60E-06 | metabolism general |
| gabT | *b2662* | 3.25 | OP25 | 1.20E-03 | metabolism general |
| gabD | *b2661* | 4.20 | OP25 | 2.21E-03 | metabolism general |
| pstS | *b3728* | 3.62 |  | 7.38E-04 | metabolism general |
| b2659 | *b2659* | 4.19 | OP25 | 7.98E-05 | no |
| ygaF | *b2660* | 4.30 | OP25 | 8.05E-03 | no |
| hdeB | *b3509* | 6.87 | OP26 | 4.13E-04 | no |
| yieJ | *b3717* | 3.33 | OP29 | 3.14E-03 | no |
| yieI | *b3716* | 6.69 | OP29 | 9.48E-03 | no |
| yjcH | *b4068* | 4.87 | OP33 | 1.88E-03 | no |
| ybdB | *b0597* | 3.50 | OP8 | 1.09E-03 | no |
| yjeF | *b4167* | 2.00 |  | 1.13E-02 | no |
| msyB | *b1051* | 2.01 |  | 1.61E-02 | no |
| yjeE | *b4168* | 2.02 |  | 4.70E-02 | no |
| yaiA | *b0389* | 2.09 |  | 3.70E-02 | no |
| ycjL | *b1298* | 2.10 |  | 3.10E-03 | no |
| yhjJ | *b3527* | 2.10 |  | 3.27E-03 | no |
| b1452 | *b1452* | 2.16 |  | 6.21E-03 | no |
| yaeR | *b0187* | 2.20 |  | 2.04E-02 | no |
| yigI | *b3820* | 2.35 |  | 3.94E-03 | no |
| yqeF | *b2844* | 2.35 |  | 9.35E-03 | no |
| yafH | *b0221* | 2.37 |  | 1.90E-02 | no |
| yidS | *b3690* | 2.62 |  | 2.58E-03 | no |
| nrdI | *b2674* | 3.66 |  | 8.43E-04 | no |
| insA1 | *b0022* | 4.60 |  | 5.68E-03 | no |
| yjgK | *b4252* | 4.64 |  | 4.84E-04 | no |
| insA2 | *b0265* | 5.48 |  | 7.96E-04 | no |
| yjcD | *b4064* | 9.66 |  | 3.63E-04 | no |
| purN | *b2500* | 7.67 | OP22 | 1.70E-03 | nucleotide |
| purM | *b2499* | 8.13 | OP22 | 1.10E-03 | nucleotide |
| purD | *b4005* | 7.67 | OP31 | 4.78E-05 | nucleotide |
| purH | *b4006* | 13.62 | OP31 | 1.04E-05 | nucleotide |
| purF | *b2312* | 8.27 | OP51 | 3.48E-04 | nucleotide |
| purK | *b0522* | 5.82 | OP6 | 3.64E-04 | nucleotide |
| purE | *b0523* | 6.19 | OP6 | 1.11E-04 | nucleotide |
| deoA | *b4382* | 2.02 |  | 1.02E-02 | nucleotide |
| cytR | *b3934* | 2.05 |  | 4.63E-04 | nucleotide |
| udp | *b3831* | 2.64 |  | 2.17E-02 | nucleotide |
| ndk | *b2518* | 3.13 |  | 3.31E-04 | nucleotide |
| purC | *b2476* | 4.70 |  | 5.63E-04 | nucleotide |
| purL | *b2557* | 7.95 |  | 6.77E-05 | nucleotide |
| purT | *b1849* | 16.92 |  | 1.76E-05 | nucleotide |
| carB | *b0033* | 2.03 | OP1 | 8.28E-03 | nucleotide/amino acid |
| carA | *b0032* | 2.50 | OP1 | 4.98E-03 | nucleotide/amino acid |
| cycA | *b4208* | 3.30 |  | 1.15E-04 | nucleotide/amino acid |
| pssR | *b3763* | 2.10 |  | 2.42E-03 | phospholipid |
| fimI | *b4315* | 2.71 | OP35 | 2.20E-03 | pilus |
| fimA | *b4314* | 3.11 | OP35 | 1.53E-03 | pilus |
| crl | *b0240* | 3.54 |  | 3.89E-03 | pilus |
| rmf | *b0953* | 3.84 |  | 4.33E-04 | ribosome/chaperoning |
| yjcG | *b4067* | 2.92 | OP33 | 2.08E-03 | transport |
| ytfQ | *b4227* | 2.47 |  | 8.99E-04 | transport |
| ydhC | *b1660* | 2.51 |  | 3.61E-03 | transport |
| leuT | *b3798* | 2.40 |  | 1.01E-02 | tRNA |
| ***More highly expressed in* DH5α** | | | |  |  |
| eutC | *b2440* | 3.04 | OP21 | 1.05E-02 | amines |
| b2439 | *b2439* | 2.36 | OP21 | 1.79E-03 | amines |
| gatD | *b2091* | 64.44 | OP19 | 2.00E-05 | carbon |
| gatC | *b2092* | 32.99 | OP19 | 4.25E-05 | carbon |
| gatB | *b2093* | 17.21 | OP19 | 3.14E-06 | carbon |
| gatR | *b2090* | 3.33 | OP19 | 4.55E-04 | carbon |
| gatA | *b2094* | 2.47 | OP19 | 1.87E-02 | carbon |
| slyB | *b1641* | 2.07 |  | 3.02E-03 | cell structure |
| cheB | *b1883* | 20.78 | OP14 | 9.25E-04 | chemotaxis |
| cheY | *b1882* | 13.64 | OP14 | 2.34E-04 | chemotaxis |
| tar | *b1886* | 57.21 | OP15 | 9.55E-05 | chemotaxis |
| tap | *b1885* | 18.87 | OP15 | 4.29E-05 | chemotaxis |
| cheA | *b1888* | 51.81 | OP16 | 1.86E-05 | chemotaxis |
| cheW | *b1887* | 20.76 | OP16 | 1.13E-05 | chemotaxis |
| tsr | *b4355* | 25.94 |  | 1.10E-04 | chemotaxis |
| trg | *b1421* | 2.97 |  | 1.07E-03 | chemotaxis |
| aer | *b3072* | 5.90 |  | 6.22E-04 | chemotaxis/carbon |
| napF | *b2208* | 6.83 | OP20 | 1.35E-02 | energy |
| napD | *b2207* | 2.21 | OP20 | 1.37E-02 | energy |
| narH | *b1225* | 3.35 |  | 9.27E-04 | energy |
| glpD | *b3426* | 2.12 |  | 3.40E-03 | energy |
| pflA | *b0902* | 2.09 |  | 1.70E-03 | energy |
| yagE | *b0268* | 11.44 | OP2 | 8.27E-05 | extrachromosomal |
| yagF | *b0269* | 2.26 | OP2 | 2.49E-02 | extrachromosomal |
| yagN | *b0280* | 39.63 | OP3 | 1.83E-04 | extrachromosomal |
| intF | *b0281* | 14.68 | OP3 | 9.20E-03 | extrachromosomal |
| nmpC | *b0553* | 1496.12 |  | 8.61E-05 | extrachromosomal |
| ykfB | *b0250* | 5.72 |  | 1.02E-02 | extrachromosomal |
| b2450 | *b2450* | 5.17 |  | 1.19E-03 | extrachromosomal |
| ybcU | *b0557* | 3.73 |  | 3.07E-03 | extrachromosomal |
| flu | *b2000* | 3.32 |  | 2.52E-02 | extrachromosomal |
| trs5_8 | *b2192* | 2.72 |  | 5.30E-03 | extrachromosomal |
| yagI | *b0272* | 2.03 |  | 2.09E-02 | extrachromosomal |
| flgI | *b1080* | 120.60 | OP10 | 8.30E-05 | flagellum |
| flgF | *b1077* | 28.98 | OP10 | 5.72E-05 | flagellum |
| flgB | *b1073* | 24.32 | OP10 | 6.52E-04 | flagellum |
| flgH | *b1079* | 23.57 | OP10 | 4.36E-06 | flagellum |
| flgD | *b1075* | 18.90 | OP10 | 1.24E-04 | flagellum |
| flgM | *b1071* | 17.23 | OP10 | 8.02E-05 | flagellum |
| flgE | *b1076* | 16.71 | OP10 | 1.91E-04 | flagellum |
| flgG | *b1078* | 15.97 | OP10 | 1.81E-04 | flagellum |
| flgN | *b1070* | 14.84 | OP10 | 1.14E-04 | flagellum |
| flgC | *b1074* | 14.73 | OP10 | 5.17E-05 | flagellum |
| flgA | *b1072* | 8.58 | OP10 | 5.99E-04 | flagellum |
| flgJ | *b1081* | 6.14 | OP10 | 7.27E-05 | flagellum |
| flhA | *b1879* | 25.53 | OP13 | 2.84E-04 | flagellum |
| flhE | *b1878* | 10.01 | OP13 | 5.81E-06 | flagellum |
| flhB | *b1880* | 5.42 | OP13 | 4.72E-04 | flagellum |
| motB | *b1889* | 45.32 | OP16 | 2.26E-04 | flagellum |
| motA | *b1890* | 32.14 | OP16 | 2.48E-03 | flagellum |
| fliD | *b1924* | 109.26 | OP17 | 2.89E-06 | flagellum |
| fliS | *b1925* | 29.40 | OP17 | 2.19E-05 | flagellum |
| fliZ | *b1921* | 21.44 | OP17a | 1.40E-05 | flagellum |
| fliA | *b1922* | 17.54 | OP17a | 6.98E-05 | flagellum |
| fliY | *b1920* | 2.20 | OP17a | 3.63E-03 | flagellum |
| fliJ | *b1942* | 26.34 | OP18 | 2.03E-05 | flagellum |
| fliH | *b1940* | 10.99 | OP18 | 3.27E-04 | flagellum |
| fliI | *b1941* | 10.12 | OP18 | 3.37E-05 | flagellum |
| fliK | *b1943* | 9.85 | OP18 | 2.69E-04 | flagellum |
| fliF | *b1938* | 9.36 | OP18 | 6.92E-05 | flagellum |
| fliG | *b1939* | 8.33 | OP18 | 8.47E-04 | flagellum |
| fliN | *b1946* | 16.73 | OP50 | 7.93E-06 | flagellum |
| fliM | *b1945* | 13.54 | OP50 | 1.89E-06 | flagellum |
| fliP | *b1948* | 10.36 | OP50 | 1.92E-04 | flagellum |
| fliQ | *b1949* | 9.49 | OP50 | 9.64E-03 | flagellum |
| fliC | *b1923* | 104.97 |  | 1.37E-06 | flagellum |
| flhC | *b1891* | 5.13 |  | 1.88E-04 | flagellum |
| fliE | *b1937* | 2.78 |  | 8.02E-03 | flagellum |
| flgK | *b1082* | 32.62 | OP10 | 5.94E-05 | flagellum/chaperone |
| fliT | *b1926* | 39.37 | OP17 | 7.00E-05 | flagellum/chaperone |
| flgL | *b1083* | 57.36 |  | 2.58E-04 | flagellum/chaperone |
| hsdR | *b4350* | 15.11 |  | 4.41E-03 | endonuclease R |
| ycgR | *b1194* | 33.64 |  | 4.16E-05 | information |
| ftn | *b1905* | 4.29 |  | 3.69E-03 | information |
| yahA | *b0315* | 3.50 |  | 4.89E-02 | information |
| ykgA | *b0300* | 2.73 |  | 2.03E-03 | information |
| sodB | *b1656* | 2.73 |  | 1.82E-03 | information |
| galU | *b1236* | 2.63 |  | 3.75E-02 | M/K antigen |
| aceA | *b4015* | 5.65 | OP32 | 2.38E-03 | metabolism general |
| aceK | *b4016* | 2.52 | OP32 | 7.29E-03 | metabolism general |
| modB | *b0764* | 3.16 | OP9 | 2.11E-03 | metabolism general |
| modA | *b0763* | 3.11 | OP9 | 9.43E-04 | metabolism general |
| modC | *b0765* | 2.56 | OP9 | 7.05E-04 | metabolism general |
| narK | *b1223* | 3.13 |  | 4.39E-02 | metabolism general |
| ribB | *b3041* | 2.75 |  | 3.35E-03 | metabolism general |
| ppsA | *b1702* | 2.74 |  | 2.21E-03 | metabolism general |
| oppC | *b1245* | 2.81 | OP11 | 2.07E-03 | murein |
| oppB | *b1244* | 2.38 | OP11 | 1.98E-02 | murein |
| oppA | *b1243* | 2.37 | OP11 | 1.43E-02 | murein |
| oppD | *b1246* | 2.35 | OP11 | 3.42E-03 | murein |
| yhjH | *b3525* | 12.28 |  | 1.98E-04 | no |
| ykgE | *b0306* | 17.58 | OP4 | 8.99E-05 | no |
| ykgF | *b0307* | 6.32 | OP4 | 2.38E-02 | no |
| ykgG | *b0308* | 4.04 | OP4 | 4.60E-03 | no |
| b1904 | *b1904* | 21.65 |  | 2.74E-03 | no |
| b1742 | *b1742* | 7.39 |  | 3.30E-03 | no |
| b1045 | *b1045* | 6.73 |  | 1.18E-04 | no |
| b1760 | *b1760* | 5.64 |  | 2.64E-04 | no |
| yjcZ | *b4110* | 4.99 |  | 2.30E-02 | no |
| b1826 | *b1826* | 2.18 |  | 1.44E-03 | no |
| guaB | *b2508* | 3.02 | OP23 | 1.04E-03 | nucleotide |
| guaA | *b2507* | 2.45 | OP23 | 1.95E-02 | nucleotide |
| codA | *b0337* | 13.11 |  | 4.23E-03 | nucleotide |
| feoA | *b3408* | 2.56 |  | 3.86E-02 | transport |
| yciD | *b1256* | 2.41 |  | 3.30E-02 | transport |

OP indicates location of genes in same operon. Operon numbering is arbitrary solely intended to indicate the membership of genes to certain operons.
