# Supplementary material for: Gene expression analysis indicates extensive genotype-specific crosstalk between the conjugative F-plasmid and the E. coli chromosome
Source: BMC Microbiol. 2006 Sep 18;6:80. doi: 10.1186/1471-2180-6-80 (PMC1590023; doi:10.1186/1471-2180-6-80)
Supplement: Additional File 2 — Bacterial/F plasmid interaction genes. List of genes that show an interaction effect between host genotype and F plasmid. [file 1471-2180-6-80-S2.doc]

**Additional file 2**

“Bacterial/F-plasmid interaction effect” genes

| Gene | b no. | fold change DH5α | fold change MG1655 | ANOVA interaction P-value | Operon no. | functional class |
| --- | --- | --- | --- | --- | --- | --- |
|  |  |  |  |  |  |  |
| Set I |  |  |  |  |  |  |
| *aroA* | b0908 | -2.01 |  | 3.84E-02 | **7** | amino acid |
| *lysU* | b4129 | -2.89 |  | 1.55E-02 |  | amino acid |
| *tdcB* | b3117 | 2.96 |  | 2.13E-03 | 2 | amino acid (thr) |
| *tdcD* | b3115 | 4.45 |  | 1.00E-02 | 2 | amino acid (thr) |
| *tdcE* | b3114 | 2.86 |  | 1.81E-02 | 2 | amino acid (thr) |
| *glcB* | b2976 | -3.38 |  | 1.42E-02 | **1** | carbon |
| *ygcY* | b2788 | 2.36 |  | 7.55E-03 |  | carbon |
| *fucU* | b2804 | 2.18 |  | 2.69E-02 |  | carbon |
| *uhpT* | b3666 | 5.05 |  | 1.86E-04 |  | carbon |
| *araF* | b1901 | 2.10 |  | 1.32E-02 |  | carbon |
| *mglA* | b2149 | 3.26 |  | 1.49E-03 | 5 | carbon |
| *mglC* | b2148 | 3.23 |  | 4.89E-03 | 5 | carbon |
| *mglB* | b2150 | 2.36 |  | 5.29E-03 | 5 | carbon/chaperone |
| *ygiM* | b3055 | -2.43 |  | 5.65E-03 |  | cell structure |
| *dnaK* | b0014 | -2.28 |  | 3.10E-02 |  | chaperone |
| *hslV* | b3932 | -2.13 |  | 6.81E-03 |  | chaperone |
| *aldH* | b1300 | -11.51 |  | 1.72E-02 |  | energy |
| *glcF* | b2978 | -2.67 |  | 5.23E-03 | **1** | metabolism general |
| *glgB* | b3432 | -2.24 |  | 9.40E-03 |  | metabolism general |
| *glgC* | b3430 | -2.11 |  | 4.75E-02 |  | metabolism general |
| *speA* | b2938 | 2.83 |  | 2.52E-02 |  | metabolism general |
| *poxB* | b0871 | -3.05 |  | 3.69E-02 |  | metabolism general |
| *b0830* | b0830 | -3.36 |  | 2.14E-03 | **6** | transport |
| *b2420* | b2420 | -3.11 |  | 3.32E-03 |  | unknown |
| *ybgL* | b0713 | -2.34 |  | 9.58E-04 |  | unknown |
|  |  |  |  |  |  |  |
| Set II |  |  |  |  |  |  |
|  |  |  |  |  |  |  |
| *ilvL* | b3766 |  | -2.99 | 3.98E-02 |  | amino acid |
| *wrbA* | b1004 |  | -2.94 | 1.52E-02 |  | amino acid |
| *aroK* | b3390 |  | 2.01 | 4.10E-02 |  | amino acid |
| *pfkA* | b3916 |  | -2.02 | 1.06E-02 |  | carbon |
| *b1627* | b1627 |  | 2.17 | 3.78E-02 |  | cell structure |
| *yfhE* | b2527 |  | 2.85 | 1.39E-03 | **8** | chaperone |
| *nirB* | b3365 |  | -4.68 | 6.44E-03 |  | energy |
| *nrfA* | b4070 |  | -2.40 | 5.88E-03 |  | energy |
| *fdnH* | b1475 |  | -2.20 | 3.27E-03 |  | energy |
| *narJ* | b1226 |  | -5.20 | 4.87E-02 |  | energy/chaperone |
| *b2001* | b2001 |  | -2.62 | 1.94E-02 |  | extrachrom |
| *fliO* | b1947 |  | 2.21 | 2.59E-02 |  | flagellum |
| *rnpA* | b3704 |  | 3.20 | 9.96E-03 | 4 | information |
| *b2531* | b2531 |  | 2.34 | 1.23E-02 | **9** | information |
| *rob* | b4396 |  | -2.40 | 1.17E-02 |  | information |
| *lgt* | b2828 |  | 2.93 | 5.07E-03 |  | information |
| *trmA* | b3965 |  | 2.16 | 9.56E-03 |  | information |
| *yidW* | b3695 |  | -2.07 | 1.94E-02 |  | information |
| *yjjM* | b4357 |  | -4.49 | 1.22E-02 |  | information |
| *araC* | b0064 |  | -2.30 | 4.17E-02 |  | information |
| *yaeG* | b0162 |  | -3.01 | 4.37E-03 |  | information |
| *sseA* | b2521 |  | -2.37 | 1.14E-03 |  | metabolism general |
| *mreD* | b3249 |  | 2.29 | 3.16E-02 |  | murein |
| *ycjI* | b1326 |  | -2.11 | 1.83E-02 |  | murein |
| *pepT* | b1127 |  | -2.84 | 4.44E-02 |  | murein? |
| *purU* | b1232 |  | 2.48 | 7.06E-03 |  | nucleotide |
| *pyrD* | b0945 |  | 2.97 | 2.92E-03 |  | nucleotide |
| *rpmH* | b3703 |  | 2.94 | 3.48E-02 | 4 | ribosome |
| *rpsV* | b1480 |  | -2.52 | 4.82E-02 |  | ribosome |
| *yfiA* | b2597 |  | -12.45 | 5.51E-03 |  | ribosome |
| *rsuA* | b2183 |  | 3.50 | 1.74E-02 |  | ribosome |
| *yrbG* | b3196 |  | 3.87 | 1.64E-02 |  | transport |
| *ybeA* | b0636 |  | 2.73 | 3.22E-03 | 3 | unknown |
| *ybeB* | b0637 |  | 3.42 | 1.48E-02 | 3 | unknown |
| *b1044* | b1044 |  | 2.41 | 1.21E-02 |  | unknown |
| *b1832* | b1832 |  | 2.08 | 4.18E-02 |  | unknown |
| *yfiH* | b2593 |  | 2.27 | 1.19E-02 |  | unknown |
| *yidQ* | b3688 |  | -2.12 | 2.64E-02 |  | unknown |
| *yjfN* | b4188 |  | -3.30 | 2.93E-03 |  | unknown |
| *yjfO* | b4189 |  | -6.65 | 1.08E-03 |  | unknown |
| *b1643* | b1643 |  | 9.55 | 1.86E-03 |  | unknown |
| *b1725* | b1725 |  | -5.75 | 1.99E-02 |  | unknown |
| *ybhQ* | b0791 |  | -2.04 | 5.33E-03 |  | unknown |
| *yfeA* | b2395 |  | -2.35 | 3.93E-02 |  | unknown |
| *ynaF* | b1376 |  | -3.51 | 1.49E-03 |  | unknown |
| *yrdC* | b3282 |  | 3.30 | 1.00E-02 |  | unknown |
| *yceG* | b1097 |  | 2.15 | 1.02E-02 |  | unknown |
| *ymdC* | b1046 |  | 2.01 | 5.15E-03 |  | unknown |
| *b3914* | b3914 |  | -2.30 | 4.58E-02 |  | unknown |
|  |  |  |  |  |  |  |
| Set III |  |  |  |  |  |  |
|  |  |  |  |  |  |  |
| *serC* | b0907 | -1.85 | 1.43 | 3.71E-04 | **7** | amino-acid |
| *truA* | b2318 | -1.78 | 1.30 | 6.76E-05 |  | amino-acid |
| *glcD* | b2979 | -2.33 | 1.72 | 1.27E-03 | **1** | carbon |
| *galS* | b2151 | 1.72 | -1.15 | 1.04E-04 |  | carbon |
| *melA* | b4119 | 2.79 | -1.58 | 7.12E-04 |  | carbon |
| *srlA* | b2702 | 2.23 | -4.06 | 8.14E-04 |  | carbon |
| *malX* | b1621 | 1.73 | -1.59 | 8.32E-04 |  | carbon |
| *hscA* | b2526 | -1.77 | 1.34 | 6.12E-04 | **8** | chaperone |
| *yfhF* | b2528 | -1.52 | 1.51 | 4.05E-03 | **9** | chaperone |
| *dsbC* | b2893 | -1.29 | 1.46 | 2.63E-04 |  | chaperone |
| *fdx* | b2525 | -1.53 | 1.28 | 9.64E-04 | **8** | energy |
| *frdA* | b4154 | 1.76 | -1.46 | 4.93E-04 |  | energy |
| *Protein-D* | *resD* | -10.91 | -3.34 | 3.40E-02 |  | F-plasmid |
| *rpoD* | b3067 | 1.35 | -1.36 | 2.75E-03 |  | information |
| *treB* | b4240 | 1.72 | -1.57 | 2.32E-03 |  | metabolism general |
| *upp* | b2498 | 1.61 | -1.42 | 2.01E-03 |  | nucleotide |
| *b0829* | b0829 | -1.70 | 1.40 | 4.71E-04 | **6** | transport |
| *yheN* | b3345 | -1.24 | 1.80 | 1.48E-04 |  | unknown |
| *yrfG* | b3399 | -1.45 | 1.42 | 1.70E-03 |  | unknown |
| *b1297* | b1297 | -2.84 | 2.17 | 2.42E-04 |  | unknown |
| *ucpA* | b2426 | 1.75 | -1.77 | 1.96E-03 |  | unknown |

* operons are labeled from 1 to 9. Operons labeled 2, 3, 4, 5 are strain-specific. Bold numbers indicate operons where

some members were identified as differentially affected in both backgrounds (Set III) and in either DH5α (Set I) or MG1655 (Set II).

Negative values indicate down-regulation in F-plasmid free cells.
